# The Endocrine Role of Estrogens on Human Male Skeleton

**DOI:** 10.1155/2015/165215

**Published:** 2015-03-19

**Authors:** Vincenzo Rochira, Elda Kara, Cesare Carani

**Affiliations:** ^1^Unit of Endocrinology, Department of Biomedical, Metabolic and Neural Sciences, University of Modena and Reggio Emilia, Via P. Giardini 1355, 41126 Modena, Italy; ^2^Azienda USL di Modena, Nuovo Ospedale Civile Sant'Agostino Estense (NOCSAE), Via P. Giardini 1355, 41126 Modena, Italy

## Abstract

Before the characterization of human and animal models of estrogen deficiency, estrogen action was confined in the context of the female bone. These interesting models uncovered a wide spectrum of unexpected estrogen actions on bone in males, allowing the formulation of an estrogen-centric theory useful to explain how sex steroids act on bone in men. Most of the principal physiological events that take place in the developing and mature male bone are now considered to be under the control of estrogen. Estrogen determines the acceleration of bone elongation at puberty, epiphyseal closure, harmonic skeletal proportions, the achievement of peak bone mass, and the maintenance of bone mass. Furthermore, it seems to crosstalk with androgen even in the determination of bone size, a more androgen-dependent phenomenon. At puberty, epiphyseal closure and growth arrest occur when a critical number of estrogens is reached. The same mechanism based on a critical threshold of serum estradiol seems to operate in men during adulthood for bone mass maintenance via the modulation of bone formation and resorption in men. This threshold should be better identified in-between the ranges of 15 and 25 pg/mL. Future basic and clinical research will optimize strategies for the management of bone diseases related to estrogen deficiency in men.

## 1. Introduction

In the human male, testosterone (T) and estradiol (E_2_) are the main circulating sex steroids acting on bone tissue. The first is produced from the Leydig cells in the testis, while the latter derives from the aromatization of the androgens by means of the enzymatic complex of aromatase [1]. Aromatase is a cytochrome P450 enzyme encoded by the* CYP19A1* gene that plays a key role in estrogen biosynthesis: it catalyzes the conversion of Δ^4^-androstenedione into estrone and that of T into E_2_ [2, 3]. Aromatase is widely expressed in a large number of tissues such as testis (Sertoli and Leydig cells), ovary (granulosa cells and luteal corpus), brain (including hypothalamus), hair follicles, and fibroblasts [2, 3]. Adipose tissue also expresses aromatase and it constitutes an important source of estrogens, especially in men [1–4]. In men, in fact, E_2_ is mainly produced by the testis and secondarily by adipose tissue [2–4]. Biological actions of estrogens are mediated by their receptor (ER) that belongs to the nuclear receptors family, and, to date, two different ERs have been identified: ER-*α* and ER-*β* [5]. A further nongenomic pathway of estrogen action has been described probably involving a plasma membrane interaction of the ER [6, 7].

Animal [8, 9] and human [10, 11] models of male congenital estrogen deficiency offered a new scenario useful for better understanding estrogen effects on male bone as well as several organs and tissues in men [12–14]. All these physiological actions of estrogens in men remained overlooked for a long time (see [13] for review). In the past, estrogen was also erroneously considered indispensable for blastocyst implantation [15] and congenital estrogen defects are supposed to be incompatible with life [10, 11]. The discovery of the first cases of congenital estrogen defects in humans allowed understanding that aromatase deficiency is due to mutations of the gene coding for the aromatase enzyme complex, which leads to lack of both estrogen synthesis and action, while estrogen resistance is due to mutations of the genes coding for estrogen receptors and leads to resistance to estrogen actions even in presence of circulating estrogens [10, 11].

This review will focus on the role of estrogens on human male bone according to all skeletal physiological events that occur* in vivo* in different life stages in men. The rise of T and E_2_ in men at puberty progressively exposes bone to sex steroids, thus allowing them to act on the growing skeleton. Sex steroids modify the way through which immature bone develops in terms of size, structure, bone mineral density (BMD), and proportions till the achievement of final skeletal maturation. After the achievement of peak bone mass, estrogens continue to influence bone remodeling in adulthood, the decline of circulating E_2_ being directly correlated with bone loss from adult to aging life.

## 2. Estrogen Effects on Bone from Early to Late Puberty

Very low levels of estrogens circulate in the blood even in male children during infancy, but their real physiological significance is not known [16]. In prepubertal boys with a genital Tanner stage 1, serum E_2_ measured with the gold standard liquid chromatography tandem mass spectrometry (LC/MS/MS) starts to increase ranging from 0.5–1.0 to about 1.9 pg/mL in healthy controls and obese boys, respectively [17, 18]. Considering the developing skeleton to not be under the effects of estrogens before puberty in male fetuses and children is a good simplification, even though this is a poorly investigated field of research (Table 1).

The initial activation of the hypothalamic-pituitary-gonadal axis in male children resulting in a progressive, slow increase of sex steroids, including estrogens, characterizes the peripubertal period [17–19]. Bone exposure to low concentrations of estrogens leads to well-known estrogen-dependent bone changes in males [20, 21].

### 2.1. Effects of Estrogens on Longitudinal Skeletal Growth

The growth of long bones occurs at the growth plate, a thin layer of cartilage that separates the epiphysis from the metaphysis [22]. The growth plate consists of three distinct layers of resting (stem cell-like), proliferative and hypertrophic chondrocytes [22, 23]. The concept that sex steroids promote epiphyseal growth and maturation during puberty in both sexes was a well-known issue in endocrinology since the beginning of the last century [24–26]. This classical endocrinological theory was based on a well-distinct action of estrogen from androgen on bone. In fact, it was believed that the former leads to growth plate maturation only in women [27], while the latter leads to growth plate maturation only in men [28, 29]. As a matter of fact, the failure of epiphyseal closure and osteoporosis observed in adult men with congenital hypogonadotropic hypogonadism or with childhood onset severe T deficiency were both traditionally ascribed to insufficient bone exposure to androgen at puberty [30, 31]. Notwithstanding evidence on estrogen actions on bone maturation in men had become available since the 1980s [32–34]; this viewpoint lasted since the beginning of the 1990s when the idea that estrogen is the main sex steroid involved in male bone maturation started to advance [35] thanks to the description of the first cases of congenital defects of estrogen synthesis or action [36–40].

The clinical phenotype presented by the unique male patient described to date [36] with estrogen resistance was very close to that of men with aromatase deficiency [37, 40]. It is characterized by tall stature, a history of continuous linear growth into adulthood, unfused epiphyses, progressive genu valgum, eunuchoid proportion of the skeleton, delayed bone age, and osteoporosis. In 1997, Carani et al. [38] demonstrated that transdermal E_2_ replacement is effective in obtaining complete epiphyseal closure, final skeletal maturation, the arrest of growth in height, the increase in BMD, and peak bone mass [10, 38]. This result was subsequently replicated by many other authors in all aromatase-deficient men described so far [40–46]. On the other hand, six months of treatment with high doses of T, given before the diagnosis of aromatase deficiency in an attempt to arrest continuous linear growth, had no effects on bone age in this patient [10, 38]. Besides, E_2_ treatment was not effective in the estrogen-resistant man, as expected [36]. All these findings suggest that epiphyseal closure is an estrogen-dependent phenomenon even in males and that androgens by themselves are not effective to ensure a normal skeletal development during late pubertal stages [10, 11, 21–23] (Table 1).

**Figure 1:**
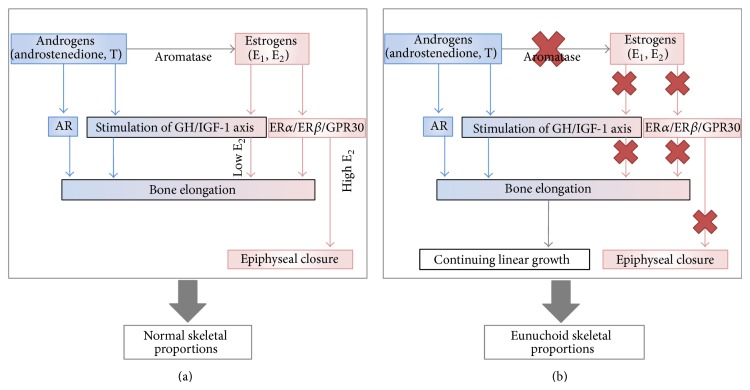
Direct and indirect effects of increasing circulating estrogens and their depletion on the growth plate (a), and effects of estrogen deprivation on bone elongation and epiphyseal status (b). AR: androgen receptor; E1: estrone; E_2_: estradiol; T: testosterone; ER-*α*: estrogen receptor alpha; ER-*β*: estrogen receptor beta; GPR30: membranous-G-protein-coupled estrogen receptor.

Later on, these findings opened the way to studies investigating the role of estrogens on bone growth and maturation not only in the context of congenital estrogen defects but also in normal boys. Serum E_2_ was found to increase simultaneously with T levels during puberty in boys and to correlate directly with chronological and skeletal age, height, weight, and pubertal stages, thus confirming the crucial role of estrogens on bone physiology at puberty even in the male [19, 21] (Table 1). In particular, estrogens seem to have a dose-dependent effect on growth plates [21, 47]: actually low doses of E_2_ stimulate ulnar growth in boys [33], while higher doses lead to an inhibition of this process of growth [20]. Recently, circulating serum E_2_ measured by LC/MS/MS resulted directly related to both the genital Tanner stage and the skeletal maturation in pubertal boys [18]. In addition, serum E_2_ was significantly higher and bone age more advanced in obese boys compared with healthy boys at the same pubertal stage [48]. The excess of adipose tissue in obese boys probably accounts for increased aromatization of androgens into estrogens and for the advancement of bone age due to the higher amounts of circulating estrogens [18, 48]. This result is in line with the well-known gender difference in the progression of skeletal maturation, which is more rapid in women than in men and parallels gender differences in the way serum E_2_ increases throughout puberty [19, 20, 33, 35]. At the beginning of puberty, when circulating E_2_ is low, the prevailing effect of E_2_ consists in the promotion of chondrocytes proliferation within the growth plate, resulting in growth plate lengthening and accelerated bone elongation (Figure 1) (Table 1). This corresponds to the increase of height velocity occurring during pubertal growth spurt that is postulated to be under estrogen control (Table 1) [21, 40, 47]. As puberty goes on, the rise in serum T ensures high E_2_ circulating levels, typical of late puberty; E_2_ inhibits chondrocyte proliferation and stimulate chondrocyte differentiation, thus inducing the progressive ossification of the growth plate and its final disappearance (Figure 1). At present, the amount of E_2_ required for shifting from the increase in length of the growth plate to its growth deceleration and final closure of the growth plate line is not known in detail. Data available in literature clearly show that no difference in serum T is present between men with idiopathic hypogonadotropic hypogonadism with fused epiphyses compared to those with unfused epiphyses [30], but no data are available in literature on serum estrogens in these rare conditions. This implies that androgen is not involved in the process of shifting from growth plate elongation to progressive growth plate thinning and final disappearance (Table 1).

**Figure 2:**
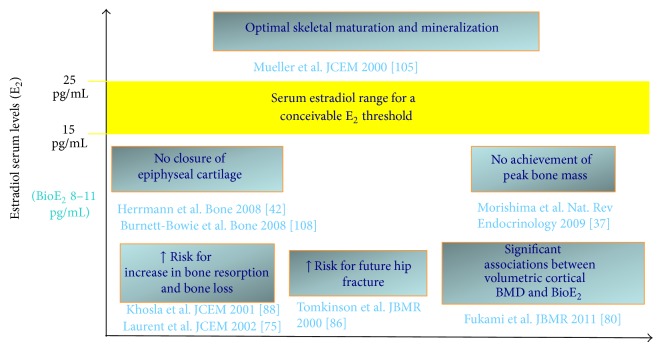
Proposed range for a critical serum estradiol threshold above which both skeletal maturation and mineralization can proceed in an optimal way. E_2_: estradiol; BioE_2_: Bioavailable estradiol.

Based on the poor compliance of an aromatase-deficient man, we tried to develop a dose-response relationship between serum E_2_ and radiological changes of the long bones in terms of bone age [45]. Due to patient's poor compliance, serum E_2_ remained below 20 pg/mL for a long time without any change of bone age and growth plate appearance at X-ray [45]. The closure of the epiphyses was obtained only several months later when E_2_ rose above 20 pg/mL and the patient was taking the right dose of transdermal E_2_ [45]. This suggests that serum E_2_ above 20 pg/mL is necessary for epiphyseal cartilage fusion [45] and that only in the case of severe estrogen deficiency the epiphyses remain still open despite the advancement of the chronological age (Figure 2). The same results can be deduced from a recent study comparing sex steroids, pubertal stage, and skeletal maturation between obese and lean boys [17]. If boys at the end of puberty (with a genital Tanner stage 5) are considered, bone age (of about 18 years on average) was greatly advanced and consistent with fused epiphyses in obese boys with a mean serum E_2_ clearly above 20 pg/mL (median 34.8 pg/mL, min–max: 25.6–41.1 pg/mL), while bone age (of about 16 years on average) was less advanced and consistent with still unfused epiphyses in lean boys with a mean serum E_2_ below 20 pg/mL (median 15.7 pg/mL, min–max: 13.2–21.0 pg/mL) [17].

The molecular mechanism through which estrogens act on the growth plate* in vivo* is still not known in detail, but recently advance on this issue has been reached [22, 23]. Several convincing evidence suggests that the growth plate width progressively decreases as a consequence of a process of senescence involving the chondrocytes, mainly in the resting zone [49]. How this senescence occurs and progresses is not known, but several mechanisms such as apoptosis, autophagy, chondrocytes differentiation into osteoblasts, and hypoxia have been proposed and are currently object of undergoing investigation by basic scientists [23]. Certainly, estrogens exert a strong effect on one or more of these pathways finally resulting in the promotion of chondrocytes involution and in the assurance of final epiphyseal closure followed by growth arrest [21–23]. What is evident is that the number of both chondrocytes and progenitor cells progressively decreases in the resting zone of the growth plate and that estrogens accelerate this process, especially when they reach a critical level [50]. Both ER-*α* and ER-*β* are expressed by human epiphyseal chondrocytes [51, 52]; moreover, the membranous-G-protein-coupled estrogen receptor, namely, GPR30 [7], is also expressed in the hypertrophic zone of human growth plate [53]. Furthermore, the fact that aromatase is also expressed by chondrocytes which are able to produce estrogens [54–56] implies that both circulating and locally produced estrogens are able to exert their actions within the growth plate via the activation of all the available estrogen transduction signaling pathways [22, 23]. Low levels of estrogens, similar to those locally produced* in vivo*, are able to promote chondrocytes proliferation and to protect them from cell death* in vitro* [57]. This mechanism might explain why low circulating and/or locally produced estrogens enhance longitudinal growth during early puberty. Conversely, the expression of the estrogen receptor GPR30 decreases dramatically during pubertal progression in humans when circulating estrogens reach the highest values typical of late puberty and longitudinal growth decelerates up to cessation [53]. GPR30 is a good candidate for explaining estrogen actions on growth plate since knock-out mice in which this receptor is disrupted do not respond to estrogen in terms of longitudinal growth deceleration and cessation [58]. ER-*β*, rather than ER-*α* [59], seems to be mainly involved in the induction of growth plate fusion in response to supraphysiological E_2_ exposure [60]. However, the complex interaction between estrogens and their receptors within the growth plate remains to be elucidated in detail. In the unique man with estrogen resistance, epiphyseal fusion did not occur at the expected time, despite high circulating E_2_ and normally functioning ER-*β* [36]. Besides, cultured cells obtained from the bone biopsy of this patient did not respond to estrogen exposure, differently from wild type cells [61, 62]. This issue is further complicated by the possible crosstalk between ER-*α* and ER-*β*. Theoretically, in fact, it is possible that the residual truncated N-terminal fragment of the disrupted ER-*α* may have acted as a negative inhibitor of ER-*β* in this patient [62, 63], thus accounting for the very delayed epiphyseal closure in this estrogen-resistant man reached at the age of 35.5 years [61].

Other hormones are good candidates for explaining growth plate proliferation, longitudinal bone growth, and growth plate involution [23]. Among them, growth hormone (GH) and insulin-like growth factor-1 (IGF-1) exert an anabolic effect on bone and are necessary for longitudinal bone growth and growth acceleration during infancy and at the time of the growth spurt, respectively [23]. The role of GH and IGF-1 on longitudinal bone growth and growth spurt could be even indirect through the well-known ability of estrogens to enhance GH and IGF-1 secretion, an event that occurs during late puberty and that concurs to accelerate growth during the pubertal spurt [10, 11, 35, 64] (Figure 1). However, longitudinal bone growth might occur and progress also independently from the GH/IGF-1 status but at a lower rate since men with aromatase deficiency continue to slowly increase their stature during adulthood, despite severe GH deficiency [65]. Accordingly, GH response to GHRH plus Arginine in four patients with aromatase deficiency was significantly lower than that in normal subjects, both before and after transdermal E_2_ replacement therapy with E_2_ [65]. In particular, E_2_ replacement did not restore normal GH secretion and IGF-1 that remained significantly lower than normal age-matched controls [65]. The fact that estrogen replacement treatment was effective on epiphyseal closure and growth arrest in all patients with aromatase deficiency [40], despite insufficient GH and IGF-1 production [65], implies that GH and IGF-1 do not play a major role in the process of growth plate closure in humans. These data suggest that a tall stature (higher than the genetic target) may be reached despite the coexistence of GH deficiency in these patients. Even though we have no data about the GH-IGF1 axis in these patients during their childhood and puberty, they were able to increase their stature during the first period of E_2_ treatment, soon before epiphyseal closure [40]. Besides, when aromatase inhibitors are administered at puberty in order to increase final height, usually a reduction of GH and IGF1 levels is observed but a benefit on final height is even obtained thanks to the epiphyseal fusion blockade and a longer time available for growth [66, 67] (Figure 2). All these data suggest that both longitudinal bone growth and a slow progressive increase in height during adulthood are possible even in presence of circulating GH and IGF-1 lower than normal on condition that epiphyseal growth plates remains open. This condition might lead to the development of tall stature [65]. Outside the context of these rare clinical conditions that help to better know how sex steroids act on the growing skeleton in healthy children and boys, GH and IGF-1 remain very important physiological determinants of growth during infancy and puberty since they ensure bone elongation and a normal height velocity [64]. Accordingly, GH deficiency represents one of the most important causes of growth retardation and (if untreated) of final short stature. Indeed, r-hGH replacement treatment is effective in restoring a normal height velocity in children and boys with GH deficiency [64].

### 2.2. Effects of Estrogens on Skeletal Proportions

In men, estrogen at puberty modulates both growth and the increase in stature in a fascinating way that allows accelerating the growth of the appendicular skeleton for a brief period only—characterized by low but detectable serum E_2_ (early puberty)—while preserving, at the same time, harmonic skeletal proportions. Accordingly, the further increase of serum estrogens triggers both epiphyseal closure and cessation of growth during mid-to-late puberty, thus avoiding the development of an altered ratio between the appendicular and the axial skeleton. Even though androgens alone are not able to induce bone maturation, they exert direct and indirect anabolic action on bone before, during, and after puberty (Figure 1). In particular, during puberty, androgens probably promote the continuous linear growth, especially at the level of long bones as substantiated by the expression of androgen receptors within the human growth plate [23, 68] and the promotion of growth sustained by androgens through the elongation of the growth plate, at least in rats [69] (Figure 1). Disproportional growth of long bones leads to eunuchoid proportions of the skeleton characterized by the prevailing length of arms and legs over the spine. Usually the eunuchoid skeleton is defined by the finding of an abnormal upper to lower segment ratio (<0.88) and by the predominance of the arm span over the patient's height (ratio > 1) [40, 47, 70]. Men with estrogen deficiency have a prolonged time available for linear growth thanks to a still open growth plate and they exhibit eunuchoid body proportions (Figure 1) [40, 47], which worsen if they are not treated with exogenous estrogens [43]. Conversely, if the onset of E_2_ treatment starts at the proper time, during early puberty, body proportions are unaffected in males with aromatase [41]. This evidence highlights the concept that estrogen rather than androgen is necessary also for a harmonic skeletal growth (Figure 1) (Table 2). As a matter of fact, normal skeletal proportions are found in patients affected by complete androgens insensitivity syndrome (CAIS) where normal-to-high E_2_ serum levels allow epiphyseal closure at the right timing, despite the absence of androgen action [71, 72]. The adult height of patients with CAIS usually corresponds, in fact, to both the calculated target and mean height of men rather than females standard [72]. All clinical conditions that lead to severe T deficiency before the completion of puberty and that are characterized by eunuchoid proportions of the skeleton [70] share the same mechanism of severe estrogen deficiency secondary to hypogonadism [73] (Figure 1). Thus, relative severe estrogen deficiency secondary to insufficient androgen production leads to eunuchoid body proportions in prepubertal male hypogonadism [70, 73], 17,20-lyase deficiency and combined 17*α*-hydroxylase and 17,20-lyase deficiency [40, 47].

### 2.3. Effects of Estrogens on Bone Mass Accrual and Attainment of Peak Bone Mass

The peak of bone mass (bone growth and BMD increase during childhood and young adulthood) determines the total amount of bone mineralized tissue available during adulthood till aging [74, 75]. Once the peak of bone mass is achieved during puberty and early adulthood (before the age of about 30 years), the bone becomes unable to reach further significant bone mass accrual. Thus, the bone mass remains stable or decreases on the basis of the balance between bone formation and resorption [74, 75]. The achievement of peak bone mass is prompted by the rise in sex steroids at puberty. Even this process was previously supposed to be under the control of estrogens in females and of androgens in males [10, 11, 76]. All the events that can negatively interfere with the achievement of peak bone mass predispose to the development of osteoporosis later in life in both sexes [74, 75]. Among them, physiological or pathological conditions that determine sex steroid deficiency result in impaired peak of bone mass, such as in the case of prepubertal hypogonadism or delayed puberty in boys and anorexia nervosa in girls [30, 31, 75].

**Figure 3:**
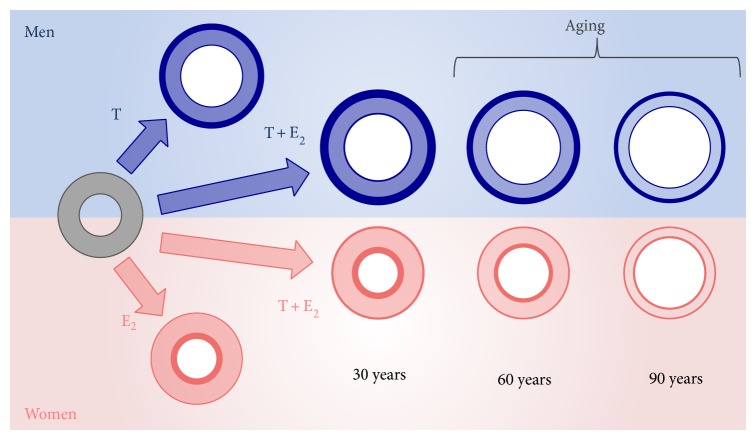
Schematic representation of the role of estrogen and androgen bone size according to gender. The effects of sex steroids on cortical and trabecular bone are represented. Bone size is reached in late puberty and early adulthood as depicted at the left of the panel where the effect of each sex steroid and their sum are shown according to gender. In men, the combined action of both T and E_2_ led to greater bone size and cortical thickness than in women. The prevailing effect of E_2_ is consistent with higher endosteal bone formation in women. Bone loss during aging occurs in a different fashion between man and women and is subordinate to the baseline conditions. Women lose more bone on the endosteal surface and in the trabecular portion of bone, while men lose mainly bone mass in the cortical bone (right side of the panel). E_2_: estradiol, T: testosterone; modified in part from figures published in [75, 77].

Probably androgens alone are not sufficient for the achievement of a normal BMD and an optimal peak of bone mass since severe osteoporosis was reported in all young adults with estrogen resistance [36] or aromatase deficiency [38–40]. The importance of estrogens for the acquisition of peak bone mass during puberty is evident by the results obtained from both human [47] and mice models [8] of estrogen deficiency (Table 1). Several mice models of estrogen deficiency have been generated and all confirmed that even in rodent estrogens mediate most of the actions exerted on bone by androgens [8, 77, 78]. In particular, the knock-out of the ER-*α* in male mice leads to the increase of trabecular bone, the reduction of cortical bone, and the decrease of longitudinal bone growth, while the knock-out of the ER-*β* does not impact cortical and trabecular bone [78]. All these data reinforce the importance of ER-*α* in male bone homeostasis [78, 79]. Studies performed on animal models, however, do not allow transposing all the results to human male bone physiology due to substantial differences in sex steroid and bone physiology among species [77]. In rodent, in fact, circulating estrogens are very low and often undetectable so that the intracrine role of estrogens prevails over that of serum E_2_ [77]. Furthermore, in rodent, sex steroids do not bind sex hormone binding globulin, and finally the process of bone maturation is different in rodent due to the absence of epiphyseal cartilage [77, 78]. The idea that estrogen is the main sex steroid involved in the acquisition of peak bone mass has been confirmed by other several data available in literature from rare models of sex steroids deficiency and from studies on pubertal boys. In men with CAIS, the peak of bone mass is only in part reduced, with intermediate values in-between those of male and female subjects [72], while men with aromatase excess syndrome display an increased BMD at the end of puberty due to high circulating estrogens throughout puberty [80]. In addition, outside the context of rare syndromes of sex steroids deficiency, several other studies involving pubertal boys clearly demonstrate that peak bone mass is under the control of estrogen even in men (Table 1) [17, 18, 48, 81–83]. During puberty, E_2_ leads to the increase of bone mass mainly by increasing BMD, especially at the level of cortical bone whereas T contributes to increase the bone size, a phenomenon that is mainly mediated by the mechanical load exerted on bone by the increasing muscle mass [18]. As far as bone geometry is concerned, E_2_ seems to be negatively associated to endosteal circumference [18] and seems to positively influence the increase of cortical thickness both at the level of radius and tibia [18]. Thus, both estrogens and androgens seem to be necessary for normal bone mass accrual during puberty. Androgens limit endosteal expansion and estrogens ensure adequate periosteal bone expansion [18] (Figure 3). The final result is a bone size greater than that of the female counterpart. Compared to females, male bone has, in fact, a larger cortical portion due to greater periosteal apposition and a larger endosteal circumference due to reduced endosteal apposition [18, 75, 77] (Figure 3). As a result of all these events, final peak bone mass is determined by the increase of BMD during puberty and early adulthood plus the remaining more slow bone accrual that continues till the 3rd decade of life and accounts for about 20% of peak bone mass [83].

## 3. Estrogen Effects on Bone during Adulthood

### 3.1. Estrogen and the Maintenance of BMD

In order to maintain a biomechanically efficient bone, the skeleton needs to continuously remodel and repair the microcracks that develop both in the trabecular and cortical bone during lifetime [75, 83]. This process of remodeling occurs in basic multicellular units (BMUs) which include osteoclasts, osteoblasts, and osteocytes [84] that act altogether by coupling bone resorption and bone formation. The balance between bone formation and bone resorption determines the maintenance (if the two processes balance out the amount of bone mass) or the loss (in the case of resorption higher than formation) of bone mass in men [74, 84]. Sex steroids exert a direct action on the BMUs and can regulate, at least in part, bone remodeling [83–85]. While this effect was traditionally ascribed to estrogens in females, in the last 20 years, several studies progressively have disclosed the same outcome in men [62, 74, 75, 83–85].

Evidence from basic research had already demonstrated a direct estrogen action on bone cells and more data, especially on biomolecular mechanisms of action, have been progressively obtained till now [84, 85]. In particular, estrogens inhibit the apoptosis of osteocytes both in trabecular and cortical bone [84, 86]. They reduce bone resorption by means of both direct and indirect effects on osteoclasts [84] and act on osteoblasts, by inhibiting their apoptosis [84, 87]. In general, estrogen regulates bone remodeling by (i) inhibiting the activation of bone remodeling and the initiation of new BMUs; (ii) reducing the number and activity of osteoclasts (i.e., inhibition of their differentiation and promotion of apoptosis) and bone resorption; and (iii) increasing the number and the activity of osteoblasts (i.e., promotion of their commitment and differentiation and inhibition of apoptosis) and bone formation [77, 79, 84, 88]. For all these reasons, bone loss in man is mainly related to relative estrogen deficiency [74, 77, 84, 88, 89], while androgens have a minor role [74, 75, 83, 84]. Accordingly, the net effect of androgens* per se* on bone mass* in vivo* is quite poor. DHT, for example, is not able to increase BMD in patients with benign prostate hyperplasia [90]. In these patients, lumbar BMD decreases of about 1.5% after 24 months of treatment as a result of the net compensation between the poor anabolic effects of high circulating DHT and its prevalent negative effect on BMD. The latter is due to the inhibition of gonadotropin secretion and the reduction of sera T and E_2_, with a reduced estrogen action on bone [90].

Several studies investigated the relationship between estrogen and BMD in men through different types of study design and all unequivocally demonstrate that estrogen action on the male bone is more determinant than androgen action [74, 75, 77, 83, 84, 89]. A normal BMD is observed in male patients with CAIS [72]. Serum E_2_ and relative estrogen deficiency resulted associated with altered bone turnover markers [91], BMD [92–98], and fracture risk [99–102]. Most of these findings were confirmed by large epidemiological longitudinal studies [92, 94, 96–101]. Altogether, these studies provide evidence that E_2_ is a better predictor of male bone health than T.

In addition, E_2_ administered to male to female transsexuals [103–105] or to men with prostate cancer [106] significantly increases BMD, despite endogenous serum T suppression. Otherwise, aromatase inhibitors lead to alterations of bone turnover markers [107] and impairment of BMD [108]. Finally, genetic studies revealed an association between ERs [109, 110] or aromatase enzyme [111, 112] polymorphisms with decreased BMD in men.

Exogenous E_2_ acts in a dose-dependent fashion on male bone since it restores a normal BMD in aromatase-deficient men given at a dose that ensures stable serum E_2_ levels within the normal male range [40, 113, 114]. A daily dose of E_2_ lower than 20 *μ*g is usually unable to keep serum E_2_ within the normal range in these patients. The result is BMD worsening or failure in restoring a normal BMD in men previously treated with higher doses [113] and in naive patients [45], respectively. Similarly, in adult men without genetic diseases involving estrogen pathways, E_2_ seems to be protective for bone but only when serum levels are above a critical threshold [102]. In cohort studies, this threshold has been settled in-between 15 and 20 pg/mL [115]. By studying the effects of estrogen treatment in an aromatase-deficient man [45], this threshold has been more precisely determined as being around 16 pg/mL (Figure 2). This value has been also confirmed by studies on fracture risk in older men (Figure 2) [102]. Recently Khosla et al. stated that a serum E_2_ level at least above 25 pg/mL is certainly protective for bone in men (Figure 2) [88]. All these data suggest that serum E_2_ levels of 20 pg/mL or above are needed for optimal skeletal maturation and achievement of optimal peak bone mass [116]. This threshold is very close to that required also for the epiphyseal closure (Figure 2) [45, 116].

The concept that estrogens act on bone only when a specific amount is reached is well established, whereas the precise threshold value remains to be settled. Differences in study design and overall in methods used for estrogen assays might explain the discrepancy between values obtained from different studies. In the future, the wide use of the gold standard methods (LC/MS/MS) for the measurement of estrogen in serum will provide more reliable information on the exact threshold value: the latter will probably fall within the suggested range of 15–25 pg/mL (Figure 2). Furthermore, future studies will be of help in disclosing if individual, genetic differences in estrogen sensitivity might influence the amount of estrogens needed for a full estrogen action on bone.

In conclusion, clinical and basic research demonstrate that E_2_ is the main sex steroid required for bone homeostasis in men [74, 77, 83, 84, 88, 89].

### 3.2. Estrogens and Bone Size

Bone size exhibits evident gender differences that are ascribed mainly to sex steroids actions on bone [83, 117, 118]. Estrogen controls the final length of long bones by acting on epiphyseal closure (see the paragraph above for details). The length of long bones is greater in men than in women. This could be once again the effect of the more rapid increase of estrogens at puberty in women which is responsible for an anticipated epiphyseal closure and growth cessation [17, 18, 48]. Actually, bone mass and strength are greater in men than in women; probably these differences are due to different length and bone structure among the two sexes (Figure 3) [76]. Bone size, in fact, is larger in men than in women mostly as a consequence of a wider width of bone, especially of its cortical portion (Figure 3) [75, 77, 115, 118, 119]. The enlargement of the periosteum involves the appendicular skeleton, and it mainly occurs from puberty to the 3rd decade of life [75]. In men, this process leads to continuous increase of the cortical thickness (Figure 3) [119–121]. Conversely, in females, this process ceases earlier and does not continue during young adulthood [119–121]. In addition, the very high amount of estrogen in women is responsible mainly for endosteal bone formation. The final result is that adult females have smaller cortical bone portion and a shorter endosteal circumference than males (Figure 3) [75, 77, 115–121].

It has been suggested that the different levels of circulating androgens between the two sexes can explain this sexual dimorphism in bone structure. As the final pathways of sex steroids actions in bone are the same in the two sexes, this sexual dimorphism in bone size probably comes from indirect actions of sex steroids on tissues different from bone. With this in view, a possible role of T in establishing bone size can be explained by sex differences in muscle mass [122] which start to appear at the time of puberty [48]. Muscle mass is androgen-dependent [122] and greater muscle mass can exert greater mechanical action on bone [123], thus resulting in increased bone size and bone mass [75]. Obese boys, for example, have larger muscle size and larger bones in the legs if compared with lean boys at the same pubertal stage [48]. Furthermore, when T is administered to ovariectomized female-to-male transsexuals, both muscle and skeletal mass change and the final musculoskeletal system resembles that of the male counterpart [124].

The old view postulated that androgen stimulates periosteal expansion in men, whereas estrogen inhibits periosteal apposition in women. The finding of significant periosteal expansion prompted by estrogen treatment in a boy with aromatase deficiency [41] suggests a more complex cross-talk between these two hormones within the bone periosteal surface [125]. Estrogens might exert a permissive action on androgens, thus facilitating and promoting their anabolic effect on the periosteum; this event does not take place or is minimized when estrogens are lacking or are below the normal range (Figure 3) [125]. Lesson from rare models of estrogen or androgen deficiency seems to confirm this hypothesis. In fact, bone size has a female appearance in XY patients with CAIS [126, 127]. Accordingly, Vandenput and colleagues [128] studied the role of the androgen receptor in the skeletal homeostasis of androgen-resistant, testicular feminized, male mice and observed that bone size and bone formation at the periosteal surface depend also on a functional androgen receptor. The mechanism consists in a permissive estrogen role on androgens and seems to operate also on trabecular bone homeostasis [114]. This permissive estrogen action, however, needs to be confirmed and better clarified on a molecular point of view.

In conclusion, the role of each sex steroid on bone size and on gender difference is a complex phenomenon that involves also other endocrine systems like the GH/IGF-1 and that needs still to be better clarified.

## 4. Estrogen Effects on Bone While Aging

The progressive decline of T that usually occurs with advancing age might result in a corresponding decrease of circulating estrogens in men [74, 98, 102, 129]. Relative estrogen deficiency in elderly men with low T is common and has been clearly demonstrated in most of the longitudinal studies on sex steroids in older men [98, 101, 102, 129]. There is, however, the possibility that both androgens and estrogens do not decline with advancing age, especially in older men in a good health status [130]. A mild-to-severe reduction of circulating T in older men who develop age-related hypogonadism is directly responsible for bone loss, but the concomitant decrease of circulating E_2_ has a main impact on bone health [14, 129]. In older men, the 70–85% of the decrease in BMD related to sex steroids decline is imputable to estrogen deficiency while only the remaining 15–30% is imputable to androgens [74, 84, 91]. Relative estrogen deficiency causes a decrease of both trabecular [83, 88, 89, 98–101] and cortical [131] bone in older men, leading to a bone structure that strongly resembles that of young aromatase-deficient men [74, 132]. Bone size, in fact, decreases at least in part as an effect of contraction of the cortical portion of the bone [75, 77, 116, 117]. However, the main changes related to aging that occur in male bone regard the bone structure. In particular, cortical porosity increases with age [75, 77, 131] while BMD decreases [75, 77, 83, 84]. BMD changes are due to the reduction of trabecular bone volume that is sustained by the thinning of the trabeculae rather than a reduction of their number, the latter being a mechanism involved in female bone aging [75, 77, 83, 84]. Another mechanism involved in male bone aging is endosteal resorption. In men, endocortical resorption is less pronounced than in women but substantially contributes to decreasing cortical cross-sectional area and consequently bone strength (Figure 3) [74, 75, 77, 83, 84, 88, 89, 98–101, 131].

To what extent the decrease in BMD due to relative estrogen deficiency contributes to the incidence of fractures in aging men with late-onset hypogonadism is still not completely clear. Men with congenital estrogen deficiency were classically considered at risk to develop fracture, but only recently a history of pathological fractures of the forearm after minimal trauma has been observed in a man with aromatase deficiency [46]. Long-term outcome concerning fractures in those rare male patients remains still not available [40]. Data obtained from older men demonstrate that E_2_ is inversely associated with BMD [92–98] and seems to predict fractures better than T [99–102]. However, not all the older men with low serum T develop relative estrogen deficiency [98, 102] and probably only men with concomitant low serum E_2_ are at high risk of fracture. Individual differences in both aromatase activity and expression probably might explain why in the presence of low serum T only a subgroup of men with late-onset hypogonadism has relative estrogen deficiency (Table 2) [74]. However, well designed studies aimed at exploring this hypothesis are still lacking. Among men with low serum T, those with concomitant estrogen deficiency should be considered to be at high risk of osteoporotic fractures (Table 2), especially when serum E_2_ is below 25 pg/mL (Figure 2). T deficiency further contributes to increasing the risk of fractures in these patients. Accordingly, muscle mass reduction that is related to androgen depletion [122] worsens BMD [123] and increases the risk of falling in elderly men [115, 133].

In conclusion, elderly male might present relative estrogen deficiency that may be implied in several age-related conditions [14] that can affect and worsen quality of life, including bone loss.

## 5. Areas of Uncertainties

Even though now the crucial role of estrogen on bone homeostasis is well established many uncertainties still remain to be clarified both in the field of basic and clinical research as well as in the field of translational endocrinology.

### 5.1. The Research Corner

Estrogen effect on male skeleton during fetal life and childhood has not yet been investigated while preliminary data during pubertal development are becoming available [16–18, 48] (Table 1). Furthermore, data on the effects of estrogen deprivation on the growing skeleton in men with congenital estrogen deficiency are scanty [40].

The exact molecular mechanism of estrogen action on the process of skeletal maturation remains to be established in details. We still do not know whether estrogens are directly involved in epiphyseal closure or whether their effect is mediated by a more complex hormonal network (endocrine and/or paracrine) including cytokines and growth factors. The lack of animal models useful to study the pathophysiology of the growth plate complicates the advancements on this issue. The models available present several limitations. The physiology of growth plate and the process of ossification of long bones in rodents are very different from those of humans [22, 23, 60]. The rabbit is closer to human but presents several significant differences [22, 23, 49–51].

While the role of circulating estrogens on bone maturation and accrual has been clarified, the contribution of intracrine estrogen production is unknown. Furthermore, the other endocrine systems and growth factors recruited by estrogen and having an important role within the bone remains to be identified.

The effects on bone of selective estrogen receptor modulators (SERMs) remain doubtful due to conflicting results available in the literature. Raloxifene, a SERM with a proven estrogen agonist action on bone with estrogen antagonist actions on other tissues, was not effective in inducing epiphyseal closure in an aromatase-deficient man after 24 months of treatment [134]. Conversely, tamoxifen, another SERM with agonist action on bone, induces permanent growth arrest through the apoptosis of chondrocytes in rats [135]. Raloxifene, however, is able to increase BMD both in aromatase-deficient men [134] and in men with prostate cancer [136, 137].

### 5.2. The Clinical Corner

About 20 years of research in the field of estrogen on bone did not lead to significant changes in clinical practice. Therapeutic strategies aiming to target estrogens for inducing changes in the male bone include aromatase inhibitors for the treatment of short stature in children and adolescents and estrogen-like compound for osteoporosis in adult and elderly men. None of these strategies, however, reached the expected results and nowadays they remain confined to the clinical research area without any real impact and extensive use in clinical practice.

Aromatase inhibitors are effective in increasing final height [67, 138] but the results in terms of centimeters reached are less than those expected if the epiphyseal closure is completely blocked. Aromatase inhibitors decelerate the advancement of bone age [67, 138], but the latter is not completely blocked as it happens in aromatase-deficient men [67, 138]. Probably even the most potent 3rd generation aromatase inhibitors are not able to completely block the enzyme [139] or, alternatively, after a first period of successful blockade, a phenomenon of escape might occur since serum E_2_ tends to progressively increase even though at a lower rate than in the placebo group [138]. This probably also accounts for the lack of reported undesired effects, such as osteoporosis, which would be expected in the case of complete blockade of the enzyme. Recently, however, Hero et al. reported vertebral deformities in boys treated with letrozole [140]. It is not clear whether severe prepubertal and pubertal estrogen deficiency might lead to skeletal deformities. Sporadic skeletal deformities (i.e., kyphosis and bilateral femoral osteonecrosis) have been observed in men with estrogen deficiency, but the cause-effect relationship with estrogen deprivation needs to be confirmed [40]. Finally, further data from clinical trials are required in order to obtain final data on safety for the use of aromatase inhibitors in boys since theoretically more adverse events are expected to occur [141, 142].

The most critical issue in clinical practice is the poor reliability of the commercially available assays. Their accuracy and reproducibility are insufficient especially in the low range of serum E_2_ typical of men [40, 74, 143]. The development of new techniques that are considered to be the gold standard, such as LC/MS/MS, is providing precise standard methodologies useful for serum estrogen measurement [143]. In the last years, these new methodologies are becoming even more widespread among clinical laboratories since they could be cost and time saving, especially in laboratories that perform a great number of assays per day. At present, serum E_2_ is not currently part of the work-up used for the clinical diagnosis and management of male osteoporosis. Several authors, however, are going to introduce this biochemical test in the clinic when the lab outcomes are accurate. Thus, we will be able to improve our knowledge about the real critical amount of circulating estrogens required to ensure bone health.

Osteoporosis still remains an overlooked and undermanaged disease in older men [74, 144], while all the advancements in the field of estrogen deficiency and bone did not result in practical, significant changes in the approach to male osteoporosis. All these uncertainties reflect the wide differences among physicians approaching estrogen deficiency in the context of osteoporosis in older men [74, 144] as well as all the difficulties in identifying a way to physiologically increase serum E_2_ without concomitantly impairing androgen production. The only practicable way remains T therapy that is able to restore both normal sera T and E_2_ with consequent beneficial effects on bone in the presence of a normal functioning enzyme [144]. However, T replacement treatment should be considered only after careful evaluation of potential benefits and disadvantages since it could be harmful especially in older men who are not in a good health status [145–147]. It should be remarked that T treatment is currently not a treatment of choice for male osteoporosis [148].

## 6. Conclusions

Several evidence support the view that estrogens are the main sex steroids involved in processes such as bone maturation, bone mass accrual, and epiphyseal closure in men. Estrogen actions on bone, especially on bone maturation, remained quite unaltered among both gender and species during evolution, thus suggesting a high degree of conservative functions for estrogen that are also confirmed by the high degree of homologies of the aromatase enzyme. Conversely, estrogen actions on other tissues and organs are determinant in ensuring gender differences (e.g., primary and secondary sexual characteristics) and in promoting sexual divergence between the two sexes during evolution. The existence of a threshold level for serum E_2_ that is necessary for ensuring skeletal maturation and adequate bone size and BMD confirms how complex the way estrogen acts on bone in men is. All these evidence contribute to make the issue of estrogen action on bone a fascinating one in the field of both basic and experimental research and encourage researches in order to find new strategies for the management and treatment of bone diseases related to estrogen deficiency.

## Figures and Tables

**Table 1:** Role of estrogens on male bone throughout lifespan.

Life stages	Effects of estrogens on bone
Fetal life	Poorly investigated/unknown

Infancy	Poorly investigated/unknown

Puberty	
Early puberty (detectable but low E_2_)	**Growth plate lengthening ** Rapid advancement of bone age Accelerated linear bone growth Increase in height velocity (growth spurt) Assurance of adequate target height attainment
Late puberty (high E_2_)	**Thinning and progressive growth plate disappearance before epiphyseal closure ** Bone maturation Advancement of skeletal maturation Progression of bone age in late puberty Epiphyseal fusion and growth arrest Achievement of final height Allowance of skeletal body proportions Achievement of adequate peak bone mass Bone size Bone length Periosteal bone apposition (crosstalk with androgens)

Adulthood	Maintenance of bone mass

Ageing	Prevention of bone loss

E_2_: estradiol.

**Table 2:** Risk of osteoporosis and fractures along with clinical manifestations according to estrogen status in elderly men.

	Elderly men with late onset hypogonadism	
Estrogenic status	**Relative estrogen deficiency** (less functioning aromatase)	**Normal circulating estrogens** (normal functioning aromatase)

Clinical phenotype	Low serum E_2_	Normal serum E_2_
	Normal E_2_/T ratio	Impaired E_2_/T ratio
	Highest gonadotropins	Less increased gonadotropins
	Severely impaired BMD	Normal to moderate BMD decrease
	Osteoporosis	Osteopenia
		Risk of developing gynecomastia

Fracture risk	**High**	**Low**

E_2_: estradiol; T: testosterone; BMD: bone mineral density.

## References

[1] MacDonald P. C., Madden J. D., Brenner P. F., Wilson J. D., Siiteri P. K. (1979). Origin of estrogen in normal men and in women with testicular feminization. *The Journal of Clinical Endocrinology & Metabolism*.

[2] Simpson E. R., Mahendroo M. S., Means G. D. (1994). Aromatase cytochrome P450, the enzyme responsible for estrogen biosynthesis. *Endocrine Reviews*.

[3] Miller W. L., Auchus R. J. (2011). The molecular biology, biochemistry, and physiology of human steroidogenesis and its disorders. *Endocrine Reviews*.

[4] Stocco C. (2012). Tissue physiology and pathology of aromatase. *Steroids*.

[5] Enmark E., Gustafsson J.-Å. (1999). Oestrogen receptors—an overview. *Journal of Internal Medicine*.

[6] Gruber C. J., Tschugguel W., Schneeberger C., Huber J. C. (2002). Mechanisms of disease: production and actions of estrogens. *The New England Journal of Medicine*.

[7] Revankar C. M., Cimino D. F., Sklar L. A., Arterburn J. B., Prossnitz E. R. (2005). A transmembrane intracellular estrogen receptor mediates rapid cell signaling. *Science*.

[8] Couse J. F., Korach K. S. (1999). Estrogen receptor null mice: what have we learned and where will they lead us?. *Endocrine Reviews*.

[9] Murata Y., Robertson K. M., Jones M. E. E., Simpson E. R. (2002). Effect of estrogen deficiency in the male: the ArKO mouse model. *Molecular and Cellular Endocrinology*.

[10] Faustini-Fustini M., Rochira V., Carani C. (1999). Oestrogen deficiency in men: where are we today?. *European Journal of Endocrinology*.

[11] Grumbach M. M., Auchus R. J. (1999). Estrogen: consequences and implications of human mutations in synthesis and action. *The Journal of Clinical Endocrinology & Metabolism*.

[12] Deroo B. J., Korach K. S. (2006). Estrogen receptors and human disease. *Journal of Clinical Investigation*.

[13] Rochira V., Granata A. R. M., Madeo B., Zirilli L., Rossi G., Carani C. (2005). Estrogens in males: what have we learned in the last 10 years?. *Asian Journal of Andrology*.

[14] Finkelstein J. S., Lee H., Burnett-Bowie S.-A. M. (2013). Gonadal steroids and body composition, strength, and sexual function in men. *The New England Journal of Medicine*.

[15] George F. W., Wilson J. D., Knobil E., Neill I. J. (1988). Sex determination and differentiation. *The Physiology of Reproduction*.

[16] Bay K., Andersson A. M., Skakkebaek N. E. (2004). Estradiol levels in prepubertal boys and girls—analytical challenges. *International Journal of Andrology*.

[17] Vandewalle S., Taes Y., Fiers T. (2014). Sex steroids in relation to sexual and skeletal maturation in obese male adolescents. *The Journal of Clinical Endocrinology and Metabolism*.

[18] Vandewalle S., Taes Y., Fiers T. (2014). Associations of sex steroids with bone maturation, bone mineral density, bone geometry, and body composition: a cross-sectional study in healthy male adolescents. *The Journal of Clinical Endocrinology and Metabolism*.

[19] Klein K. O., Martha P. M., Blizzard R. M., Herbst T., Rogol A. D. (1996). A longitudinal assessment of hormonal and physical alterations during normal puberty in boys. II. Estrogen levels as determined by an ultrasensitive bioassay. *The Journal of Clinical Endocrinology & Metabolism*.

[20] Cutler G. B. (1997). The role of estrogen in bone growth and a maturation during childhood and adolescence. *Journal of Steroid Biochemistry and Molecular Biology*.

[21] Rochira V., Balestrieri A., Faustini-Fustini M., Carani C. (2001). Role of estrogen on bone in the human male: insights from the natural models of congenital estrogen deficiency. *Molecular and Cellular Endocrinology*.

[22] Chagin A. S., Sävendahl L. (2007). Estrogens and growth: review. *Pediatric Endocrinology Reviews*.

[23] Emons J., Chagin A. S., Sävendahl L., Karperien M., Wit J. M. (2011). Mechanisms of growth plate maturation and epiphyseal fusion. *Hormone Research in Paediatrics*.

[24] Wilkins L. (1950). Endocrine relationships and their influences upon growth and development. *The Diagnosis and Treatment of Endocrine Disorders in Childhood and Adolescence*.

[25] Kuppermann H. S., Kuppermann H. S. (1963). Male endocrinology: hypogonadism in adolescent male and cryptorchidism. *Human Endocrinology*.

[26] Blizzard R. M., Thompson R. G., Baghdassarian A., Kowarski A., Migeon C. J., Rodriguez A., Grumbach M. M., Grave G. D., Mayer F. E. (1974). The interrelationship of steroids, growth hormone and other hormones on pubertal growth. *The Control of the Onset of Puberty*.

[27] Marshall W. A. (1974). Interrelationships of skeletal maturation, sexual development and somatic growth in man. *Annals of Human Biology*.

[28] Trueta J. J., Davis J., Dobbing J. (1974). The growth and development of bones and joints: orthopedic aspects. *Scientific Foundation of Pediatrics*.

[29] van der Werff ten Bosch J. J. (1977). Testosterone as growth stimulant in man. *Pharmacology and Therapeutics*.

[30] Finkelstein J. S., Klibanski A., Neer R. M., Greenspan S. L., Rosenthal D. I., Crowley W. F. (1987). Osteoporosis in men with idiopathic hypogonadotropic hypogonadism. *Annals of Internal Medicine*.

[31] Finkelstein J. S., Neer R. M., Biller B. M. K., Crawford J. D., Klibanski A. (1992). Osteopenia in men with a history of delayed puberty. *The New England Journal of Medicine*.

[32] van den Bosch J. S., Smals A. G. H., Pieters G. F. F. M., Valk I. M., Kloppenborg P. W. (1982). Instant growth inhibition by low dose oestrogens in excessively tall boys. *Acta Endocrinologica*.

[33] Caruso-Nicoletti M., Cassorla F., Skerda M., Ross J. L., Loriaux D. L., Cutler G. B. (1985). Short term, low dose estradiol accelerates ulnar growth in boys. *The Journal of Clinical Endocrinology and Metabolism*.

[34] Cutler G. B., Cassorla F. G., Ross J. L. (1986). Pubertal growth: physiology and pathophysiology. *Recent Progress in Hormone Research*.

[35] Lee P. A., Witchel S. F. (1997). The influence of estrogen on growth. *Current Opinion in Pediatrics*.

[36] Smith E. P., Boyd J., Frank G. R. (1994). Estrogen resistance caused by a mutation in the estrogen-receptor gene in a man. *The New England Journal of Medicine*.

[37] Morishima A., Grumbach M. M., Simpson E. R., Fisher C., Qin K. (1995). Aromatase deficiency in male and female siblings caused by a novel mutation and the physiological role of estrogens. *Journal of Clinical Endocrinology and Metabolism*.

[38] Carani C., Qin K., Simoni M. (1997). Effect of testosterone and estradiol in a man with aromatase deficiency. *The New England Journal of Medicine*.

[39] Bilezikian J. P., Morishima A., Bell J., Grumbach M. M. (1998). Increased bone mass as a result of estrogen therapy in a man with aromatase deficiency. *The New England Journal of Medicine*.

[40] Rochira V., Carani C. (2009). Aromatase deficiency in men: a clinical perspective. *Nature Reviews Endocrinology*.

[41] Bouillon R., Bex M., Vanderschueren D., Boonen S. (2004). Estrogens are essential for male pubertal periosteal bone expansion. *The Journal of Clinical Endocrinology and Metabolism*.

[42] Herrmann B. L., Saller B., Janssen O. E. (2002). Impact of estrogen replacement therapy in a male with congenital aromatase deficiency caused by a novel mutation in the CYP19 gene. *Journal of Clinical Endocrinology and Metabolism*.

[43] Maffei L., Murata Y., Rochira V. (2004). Dysmetabolic syndrome in a man with a novel mutation of the aromatase gene: effects of testosterone, alendronate, and estradiol treatment. *The Journal of Clinical Endocrinology and Metabolism*.

[44] Maffei L., Rochira V., Zirilli L. (2007). A novel compound heterozygous mutation of the aromatase gene in an adult man: reinforced evidence on the relationship between congenital oestrogen deficiency, adiposity and the metabolic syndrome. *Clinical Endocrinology*.

[45] Lanfranco F., Zirilli L., Baldi M. (2008). A novel mutation in the human aromatase gene: Insights on the relationship among serum estradiol, longitudinal growth and bone mineral density in an adult man under estrogen replacement treatment. *Bone*.

[46] Pignatti E., Unluhizarci K., Kartal E. (2013). Complete aromatase deficiency in four adult men: detection of a novel mutation and two known mutations in the CYP19A1 gene. *Endocrine Abstracts*.

[47] Zirilli L., Rochira V., Diazzi C., Caffagni G., Carani C. (2008). Human models of aromatase deficiency. *Journal of Steroid Biochemistry and Molecular Biology*.

[48] Vandewalle S., Taes Y., van Helvoirt M. (2013). Bone size and bone strength are increased in obese male adolescents. *The Journal of Clinical Endocrinology and Metabolism*.

[49] Weise M., De-Levi S., Barnes K. M., Gafni R. I., Abad V., Baron J. (2001). Effects of estrogen on growth plate senescence and epiphyseal fusion. *Proceedings of the National Academy of Sciences of the United States of America*.

[50] Nilsson O., Weise M., Landman E. B., Meyers I. L., Barnes K. M., Baron J. (2014). Evidence that estrogen hastens epiphyseal fusion and cessation of longitudinal bone growth by irreversibly depleting the number of resting zone progenitor cells in female rabbits. *Endocrinology*.

[51] Kusec V., Virdi A. S., Prince R., Triffitt J. T. (1998). Localization of estrogen receptor-*α* in human and rabbit skeletal tissues. *The Journal of Clinical Endocrinology & Metabolism*.

[52] Nilsson L. O., Boman A., Sävendahl L. (1999). Demonstration of estrogen receptor-*β* immunoreactivity in human growth plate cartilage. *The Journal of Clinical Endocrinology & Metabolism*.

[53] Chagin A. S., Sävendahl L. (2007). Brief report: GPR30 estrogen receptor expression in the growth plate declines as puberty progresses. *The Journal of Clinical Endocrinology and Metabolism*.

[54] Oz O. K., Millsaps R., Welch R., Birch J., Zerwekh J. E. (2001). Expression of aromatase in the human growth plate. *Journal of Molecular Endocrinology*.

[55] Sylvia V. L., Gay I., Hardin R., Dean D. D., Boyan B. D., Schwartz Z. (2002). Rat costochondral chondrocytes produce 17*β*-estradiol and regulate its production by 1*α*,25(OH)2D3. *Bone*.

[56] van der Eerden B. C. J., van Ven J. D. E., Lowik C. W. G. M., Wit J. M., Karperien M. (2002). Sex steroid metabolism in the tibial growth plate of the rat. *Endocrinology*.

[57] Chagin A. S., Chrysis D., Takigawa M., Ritzen E. M., Sävendahl L. (2006). Locally produced estrogen promotes fetal rat metatarsal bone growth; an effect mediated through increased chondrocyte proliferation and decreased apoptosis. *Journal of Endocrinology*.

[58] Windahl S. H., Andersson N., Chagin A. S. (2009). The role of the G protein-coupled receptor GPR30 in the effects of estrogen in ovariectomized mice. *American Journal of Physiology—Endocrinology and Metabolism*.

[59] Börjesson A. E., Lagerquist M. K., Windahl S. H., Ohlsson C. (2013). The role of estrogen receptor *α* in the regulation of bone and growth plate cartilage. *Cellular and Molecular Life Sciences*.

[60] Chagin A. S., Lindberg M. K., Andersson N. (2004). Estrogen receptor-*β* inhibits skeletal growth and has the capacity to mediate growth plate fusion in female mice. *Journal of Bone and Mineral Research*.

[61] Smith E. P., Specker B., Bachrach B. E. (2008). Impact on bone of an estrogen receptor-*α* gene loss of function mutation. *The Journal of Clinical Endocrinology and Metabolism*.

[62] Smith E. P., Specker B., Korach K. S. (2010). Recent experimental and clinical findings in the skeleton associated with loss of estrogen hormone or estrogen receptor activity. *Journal of Steroid Biochemistry and Molecular Biology*.

[63] Chagin A. S., Sävendahl L. (2007). Oestrogen receptors and linear bone growth. *Acta Paediatrica*.

[64] Veldhuis J. D., Roemmich J. N., Richmond E. J. (2005). Endocrine control of body composition in infancy, childhood, and puberty. *Endocrine Reviews*.

[65] Rochira V., Zirilli L., Maffei L. (2010). Tall stature without growth hormone: four male patients with aromatase deficiency. *Journal of Clinical Endocrinology and Metabolism*.

[66] Metzger D. L., Kerrigan J. R. (1994). Estrogen receptor blockade with tamoxifen diminishes growth hormone secretion in boys: evidence for a stimulatory role of endogenous estrogens during male adolescence. *The Journal of Clinical Endocrinology & Metabolism*.

[67] Hero M., Norjavaara E., Dunkel L. (2005). Inhibition of estrogen biosynthesis with a potent aromatase inhibitor increases predicted adult height in boys with idiopathic short stature: a randomized controlled trial. *The Journal of Clinical Endocrinology & Metabolism*.

[68] Nilsson O., Chrysis D., Pajulo O. (2003). Localization of estrogen receptors-*α* and -*β* and androgen receptor in the human growth plate at different pubertal stages. *Journal of Endocrinology*.

[69] Ren S. G., Malozowski S., Sanchez P., Seet D. E., Loriaux D. L., Cassorla F. (1989). Direct administration of testosterone increases rat tibial epiphyseal growth plate width. *Acta Endocrinologica*.

[70] Nieschlag E., Behre H., Nieschlag E., Behre H. (2012). Clinical use of testosterone in hypogonadism and other conditions. *Testosterone: Action, Deficiency, Substitution*.

[71] Zachmann M., Prader A., Sobel E. H. (1986). Pubertal growth in patients with androgen insensitivity: indirect evidence for the importance of estrogens in pubertal growth of girls. *The Journal of Pediatrics*.

[72] Taes Y., Lapauw B., Vandewalle S. (2009). Estrogen-specific action on bone geometry and volumetric bone density: longitudinal observations in an adult with complete androgen insensitivity. *Bone*.

[73] Trabado S., Maione L., Salenave S. (2011). Estradiol levels in men with congenital hypogonadotropic hypogonadism and the effects of different modalities of hormonal treatment. *Fertility and Sterility*.

[74] Rochira V., Balestrieri A., Madeo B., Zirilli L., Granata A. R. M., Carani C. (2006). Osteoporosis and male age-related hypogonadism: role of sex steroids on bone (patho) physiology. *European Journal of Endocrinology*.

[75] Laurent M., Antonio L., Sinnesael M. (2014). Androgens and estrogens in skeletal sexual dimorphism. *Asian Journal of Andrology*.

[76] Bonjour J.-P., Theintz G., Buchs B., Slosman D., Rizzoli R. (1991). Critical years and stages of puberty for spinal and femoral bone mass accumulation during adolescence. *The Journal of Clinical Endocrinology and Metabolism*.

[77] Vanderschueren D., Laurent M. R., Claessens F. (2014). Sex steroid actions in male bone. *Endocrine Reviews*.

[78] Vico L., Vanacker J.-M. (2010). Sex hormones and their receptors in bone homeostasis: insights from genetically modified mouse models. *Osteoporosis International*.

[79] Callewaert F., Boonen S., Vanderschueren D. (2010). Sex steroids and the male skeleton: a tale of two hormones. *Trends in Endocrinology and Metabolism*.

[80] Fukami M., Shozu M., Ogata T. (2012). Molecular bases and phenotypic determinants of aromatase excess syndrome. *International Journal of Endocrinology*.

[81] Yilmaz D., Ersoy B., Bilgin E., Gümüşer G., Onur E., Pinar E. D. (2005). Bone mineral density in girls and boys at different pubertal stages: relation with gonadal steroids, bone formation markers, and growth parameters. *Journal of Bone and Mineral Metabolism*.

[82] Lapauw B. M., Taes Y., Bogaert V. (2009). Serum estradiol is associated with volumetric BMD and modulates the impact of physical activity on bone size at the age of peak bone mass: a study in healthy male siblings. *Journal of Bone and Mineral Research*.

[83] Riggs B. L., Khosla S., Melton L. J. (2002). Sex steroids and the construction and conservation of the adult skeleton. *Endocrine Reviews*.

[84] Khosla S., Oursler M. J., Monroe D. G. (2012). Estrogen and the skeleton. *Trends in Endocrinology and Metabolism*.

[85] Manolagas S. C., O'Brien C. A., Almeida M. (2013). The role of estrogen and androgen receptors in bone health and disease. *Nature Reviews Endocrinology*.

[86] Tomkinson A., Gevers E. F., Wit J. M., Reeve J., Noble B. S. (1998). The role of estrogen in the control of rat osteocyte apoptosis. *Journal of Bone and Mineral Research*.

[87] Kousteni S., Bellido T., Plotkin L. I. (2001). Nongenotropic, sex-nonspecific signaling through the estrogen or androgen receptors: dissociation from transcriptional activity. *Cell*.

[88] Khosla S., Melton L. J., Riggs B. L. (2011). The unitary model for estrogen deficiency and the pathogenesis of osteoporosis: is a revision needed?. *Journal of Bone and Mineral Research*.

[89] Khosla S. (2010). Update on estrogens and the skeleton. *The Journal of Clinical Endocrinology & Metabolism*.

[90] Idan A., Griffiths K. A., Harwood D. T. (2010). Long-term effects of dihydrotestosterone treatment on prostate growth in healthy, middle-aged men without prostate disease: a randomized, placebo-controlled trial. *Annals of Internal Medicine*.

[91] Falahati-Nini A., Riggs B. L., Atkinson E. J., O'Fallon W. M., Eastell R., Khosla S. (2000). Relative contributions of testosterone and estrogen in regulating bone resorption and formation in normal elderly men. *The Journal of Clinical Investigation*.

[92] Greendale G. A., Edelstein S., Barrett-Connor E. (1997). Endogenous sex steroids and bone mineral density in older women and men: the Rancho Bernardo study. *Journal of Bone and Mineral Research*.

[93] Slemenda C. W., Longcope C., Zhou L., Hui S. L., Peacock M., Johnston C. C. (1997). Sex steroids and bone mass in older men. Positive associations with serum estrogens and negative associations with androgens. *The Journal of Clinical Investigation*.

[94] Amin S., Zhang Y., Sawin C. T. (2000). Association of hypogonadism and estradiol levels with bone mineral density in elderly men from the Framingham study. *Annals of Internal Medicine*.

[95] Khosla S., Melton L. J., Atkinson E. J., O'Fallon W. M. (2001). Relationship of serum sex steroid levels to longitudinal changes in bone density in young versus elderly men. *Journal of Clinical Endocrinology and Metabolism*.

[96] Szulc P., Munoz F., Claustrat B. (2001). Bioavailable estradiol may be an important determinant of osteoporosis in men: the MINOS study. *The Journal of Clinical Endocrinology & Metabolism*.

[97] Ensrud K. E., Lewis C. E., Lambert L. C. (2006). Endogenous sex steroids, weight change and rates of hip bone loss in older men: the MrOS study. *Osteoporosis International*.

[98] Ward K. A., Pye S. R., Adams J. E. (2011). Influence of age and sex steroids on bone density and geometry in middle-aged and elderly European men. *Osteoporosis International*.

[99] Barrett-Connor E., Mueller J. E., von Mühlen D. G., Laughlin G. A., Schneider D. L., Sartoris D. J. (2000). Low levels of estradiol are associated with vertebral fractures in older men, but not women: the Rancho Bernardo Study. *Journal of Clinical Endocrinology and Metabolism*.

[100] Goderie-Plomp H. W., van der Klift M., de Ronde W., Hofman A., de Jong F. H., Pols H. A. P. (2004). Endogenous sex hormones, sex hormone-binding globulin, and the risk of incident vertebral fractures in elderly men and women: the Rotterdam study. *The Journal of Clinical Endocrinology and Metabolism*.

[101] Amin S., Zhang Y., Felson D. T. (2006). Estradiol, testosterone, and the risk for hip fractures in elderly men from the Framingham Study. *The American Journal of Medicine*.

[102] Mellström D., Vandenput L., Mallmin H. (2008). Older men with low serum estradiol and high serum SHBG have an increased risk of fractures. *Journal of Bone and Mineral Research*.

[103] Reutrakul S., Ongphiphadhanakul B., Piaseu N. (1998). The effects of oestrogen exposure on bone mass in male to female transsexuals. *Clinical Endocrinology*.

[104] van Kesteren P., Lips P., Gooren L. J. G., Asscheman H., Megens J. (1998). Long-term follow-up of bone mineral density and bone metabolism in transsexuals treated with cross-sex hormones. *Clinical Endocrinology*.

[105] Mueller A., Dittrich R., Binder H. (2005). High dose estrogen treatment increases bone mineral density in male-to-female transsexuals receiving gonadotropin-releasing hormone agonist in the absence of testosterone. *European Journal of Endocrinology*.

[106] Carlström K., Stege R., Henriksson P., Grande M., Gunnarsson P. O., Pousette A. (1997). Possible bone-preserving capacity of high-dose intramuscular depot estrogen as compared to orchidectomy in the treatment of patients with prostatic carcinoma. *Prostate*.

[107] Taxel P., Kennedy D. G., Fall P. M., Willard A. K., Clive J. M., Raisz L. G. (2001). The effect of aromatase inhibition on sex steroids, gonadotropins, and markers of bone turnover in older men. *The Journal of Clinical Endocrinology & Metabolism*.

[108] Burnett-Bowie S.-A. M., McKay E. A., Lee H., Leder B. Z. (2009). Effects of aromatase inhibition on bone mineral density and bone turnover in older men with low testosterone levels. *The Journal of Clinical Endocrinology and Metabolism*.

[109] Ongphiphadhanakul B., Rajatanavin R., Chanprasertyothin S., Piaseu N., Chailurkit L. (1998). Serum oestradiol and oestrogen-receptor gene polymorphism are associated with bone mineral density independently of serum testosterone in normal males. *Clinical Endocrinology*.

[110] Khosla S., Riggs B. L., Atkinson E. J. (2004). Relationship of estrogen receptor genotypes to bone mineral density and to rates of bone loss in men. *The Journal of Clinical Endocrinology & Metabolism*.

[111] van Pottelbergh I., Goemaere S., Kaufman J. M. (2003). Bioavailable estradiol and an aromatase gene polymorphism are determinants of bone mineral density changes in men over 70 years of age. *Journal of Clinical Endocrinology and Metabolism*.

[112] Gennari L., Masi L., Merlotti D. (2004). A polymorphic *CYP19* TTTA repeat influences aromatase activity and estrogen levels in elderly men: effects on bone metabolism. *The Journal of Clinical Endocrinology and Metabolism*.

[113] Rochira V., Faustini-Fustini M., Balestrieri A., Carani C. (2000). Estrogen replacement therapy in a man with congenital aromatase deficiency: effects of different doses of transdermal estradiol on bone mineral density and hormonal parameters. *The Journal of Clinical Endocrinology and Metabolism*.

[114] Rochira V., Zirilli L., Madeo B. (2007). Skeletal effects of long-term estrogen and testosterone replacement treatment in a man with congenital aromatase deficiency: evidences of a priming effect of estrogen for sex steroids action on bone. *Bone*.

[115] Vandenput L., Ohlsson C. (2010). Sex steroid metabolism in the regulation of bone health in men. *The Journal of Steroid Biochemistry and Molecular Biology*.

[116] Khosla S. (2008). Estrogen and bone: Insights from estrogen-resistant, aromatase-deficient, and normal men. *Bone*.

[117] Seeman E. (1997). From density to structure: growing up and growing old on the surfaces of bone. *Journal of Bone and Mineral Research*.

[118] Seeman E., Delmas P. D. (2006). Bone quality—the material and structural basis of bone strength and fragility. *The New England Journal of Medicine*.

[119] Ohlsson C., Darelid A., Nilsson M., Melin J., Mellström D., Lorentzon M. (2011). Cortical consolidation due to increased mineralization and endosteal contraction in young adult men: a five-year longitudinal study. *Journal of Clinical Endocrinology and Metabolism*.

[120] Darelid A., Ohlsson C., Nilsson M., Kindblom J. M., Mellström D., Lorentzon M. (2012). Catch up in bone acquisition in young adult men with late normal puberty. *Journal of Bone and Mineral Research*.

[121] Walsh J. S., Paggiosi M. A., Eastell R. (2012). Cortical consolidation of the radius and tibia in young men and women. *The Journal of Clinical Endocrinology & Metabolism*.

[122] Bhasin S., Jasuja R., Serra C., Nieschlag E., Behre H. (2012). Androgen effects on the skeletal muscle. *Testosterone, Action, Deficiency, Substitution*.

[123] Rauch F., Bailey D. A., Baxter-Jones A., Mirwald R., Faulkner R. (2004). The “muscle-bone unit” during the pubertal growth spurt. *Bone*.

[124] van Caenegem E., Wierckx K., Taes Y. (2012). Bone mass, bone geometry, and body composition in female-to-male transsexual persons after long-term cross-sex hormonal therapy. *The Journal of Clinical Endocrinology and Metabolism*.

[125] Vanderschueren D., Venken K., Ophoff J., Bouillon R., Boonen S. (2006). Clinical review: Sex steroids and the periosteum—reconsidering the roles of androgens and estrogens in periosteal expansion. *Journal of Clinical Endocrinology and Metabolism*.

[126] Danilovic D. L. S., Correa P. H. S., Costa E. M. F., Melo K. F. S., Mendonca B. B., Arnhold I. J. P. (2007). Height and bone mineral density in androgen insensitivity syndrome with mutations in the androgen receptor gene. *Osteoporosis International*.

[127] Han T. S., Goswami D., Trikudanathan S., Creighton S. M., Conway G. S. (2008). Comparison of bone mineral density and body proportions between women with complete androgen insensitivity syndrome and women with gonadal dysgenesis. *European Journal of Endocrinology*.

[128] Vandenput L., Swinnen J. V., Boonen S. (2004). Role of the androgen receptor in skeletal homeostasis: the androgen-resistant testicular feminized male mouse model. *Journal of Bone and Mineral Research*.

[129] Wu F. C. W., Tajar A., Beynon J. M. (2010). Identification of late-onset hypogonadism in middle-aged and elderly men. *The New England Journal of Medicine*.

[130] Sartorius G., Spasevska S., Idan A. (2012). Serum testosterone, dihydrotestosterone and estradiol concentrations in older men self-reporting very good health: the healthy man study. *Clinical Endocrinology*.

[131] Vandenput L., Lorentzon M., Sundh D. (2014). Serum estradiol levels are inversely associated with cortical porosity in older men. *The Journal of Clinical Endocrinology and Metabolism*.

[132] Rochira V., Balestrieri A., Madeo B., Zirilli L., Granata A. R., Carani C. (2005). Bone loss, sex steroids and male age related hypogonadism. *Journal of Endocrinological Investigation*.

[133] Orwoll E., Lambert L. C., Marshall L. M. (2006). Endogenous testosterone levels, physical performance, and fall risk in older men. *Archives of Internal Medicine*.

[134] Zirilli L., Maffei L., Meunier P. J., Chavassieux P., Carani C., Rochira V. (2009). The effects of long-term raloxifene and estradiol treatments on bone in a patient with congenital aromatase deficiency. *Bone*.

[135] Chagin A. S., Karimian E., Zaman F., Takigawa M., Chrysis D., Sävendahl L. (2007). Tamoxifen induces permanent growth arrest through selective induction of apoptosis in growth plate chondrocytes in cultured rat metatarsal bones. *Bone*.

[136] Doran P. M., Riggs B. L., Atkinson E. J., Khosla S. (2001). Effects of raloxifene, a selective estrogen receptor modulator, on bone turnover markers and serum sex steroid and lipid levels in elderly men. *Journal of Bone and Mineral Research*.

[137] Smith M. R., Fallon M. A., Lee H., Finkelstein J. S. (2004). Raloxifene to prevent gonadotropin-releasing hormone agonist-induced bone loss in men with prostate cancer: a randomized controlled trial. *Journal of Clinical Endocrinology and Metabolism*.

[138] Mauras N., de Pijem L. G., Hsiang H. Y. (2008). Anastrozole increases predicted adult height of short adolescent males treated with growth hormone: a randomized, placebo-controlled, multicenter trial for one to three years. *The Journal of Clinical Endocrinology and Metabolism*.

[139] Løning P. E., Eikesdal H. P. (2013). Aromatase inhibition 2013: clinical state of the art and questions that remain to be solved. *Endocrine-Related Cancer*.

[140] Hero M., Mäkitie O., Kröger H., Nousiainen E., Toiviainen-Salo S., Dunkel L. (2009). Impact of aromatase inhibitor therapy on bone turnover, cortical bone growth and vertebral morphology in pre- and peripubertal boys with idiopathic short stature. *Hormone Research*.

[141] Rochira V. (2001). Aromatase inhibitors in pubertal boys: clinical implications. *The Journal of Clinical Endocrinology & Metabolism*.

[142] Geffner M. E. (2009). Aromatase inhibitors to augment height: continued caution and study required. *Journal of Clinical Research in Pediatric Endocrinology*.

[143] Rosner W., Hankinson S. E., Sluss P. M., Vesper H. W., Wierman M. E. (2013). Challenges to the measurement of estradiol: an endocrine society position statement. *The Journal of Clinical Endocrinology and Metabolism*.

[144] Madeo B., Zirilli L., Caffagni G. (2007). The osteoporotic male: overlooked and undermanaged?. *Clinical Interventions in Aging*.

[145] Basaria S., Coviello A. D., Travison T. G. (2010). Adverse events associated with testosterone administration. *The New England Journal of Medicine*.

[146] Vigen R., O'Donnell C. I., Barón A. E. (2013). Association of testosterone therapy with mortality, myocardial infarction, and stroke in men with low testosterone levels. *The Journal of the American Medical Association*.

[147] Kaufman J. M. (2014). Mortality associated to late-onset hypogonadism: reasons not to treat with testosterone?. *Journal of Clinical Endocrinology and Metabolism*.

[148] Hernlund E., Svedbom A., Ivergård M. (2013). Osteoporosis in the European Union: medical management, epidemiology and economic burden: a report prepared in collaboration with the International Osteoporosis Foundation (IOF) and the European Federation of Pharmaceutical Industry Associations (EFPIA). *Archives of Osteoporosis*.

